# Exploring the Role of Estrogens in Lizard Spermatogenesis through the Study of Clomiphene and FSH Effects

**DOI:** 10.1155/2017/4760638

**Published:** 2017-12-31

**Authors:** Mariailaria Verderame, Rosaria Scudiero, Ermelinda Limatola

**Affiliations:** Department of Biology, University Federico II, Via Mezzocannone 8, 80134 Naples, Italy

## Abstract

Spermatogenesis is a fascinating biological process aiming to generate haploid spermatozoa from diploid spermatogonia through a specific hormonal network between gonadotropins and steroids. Increasing evidence suggests that the primary female sex hormone estrogen plays an active role in this process. This research points out on the role of estrogen during lizard spermatogenesis by using three experimental approaches: (1) exposure to an analogue of nonsteroidal estrogen as Clomiphene citrate that acts both as estrogen agonist and antagonist; (2) exposure to the gonadotropin FSH; and (3) exposures to FSH followed by Clomiphene. Histological and immunohistochemical results demonstrate that in the lizard *Podarcis sicula* during the mating period, Clomiphene as well as FSH determines the breakdown of spermatogenesis and the epididymal regression, presumably through estrogens input as indirectly demonstrated by the appearance of ER*α* and vitellogenin in the liver. The ability of Clomiphene to restore the gonadal natural condition after FSH treatment is also demonstrated. Finally, data indicate that lizard testis and epididymis control their morphophysiology regulating the intracellular presence of ER*α*.

## 1. Introduction

Spermatogenesis is a finely regulated process maintained by a perfect balance between gonadotropins and sexual steroids as androgens and estrogens. In this hormonal crosstalk, gonadotropins are the prime regulators of both spermatogenesis and steroidogenesis [1]; estrogens in turn and surprisingly play a key role also in male reproduction. In the testis, LH controls testosterone production of Leydig cells, whereas FSH controls the Sertoli cell functions, including the production of estrogens and of many locally active factors orchestrating germ cell development [2–6]. The ability of the testis to synthesize estrogens has been widely recognized, as well as the capacity of the testis to react to the estrogen stimulation through the presence of estrogen receptors (ERs) [7–10]. Many studies have been performed in knockout mice lacking functional ERs or aromatase; these animals show testicular and epididymal atrophy and other reproductive disruptions leading to infertility [11–14]. In rat, when ERs are blocked by the antagonist ICI 182,780 for 100–150 days, testicular atrophy and infertility occur [15]. On the other hand, too much estrogen in adults downregulates gonadotropin synthesis and alters the entire hormonal milieu causing the impairment of spermatogenesis and the apoptosis of the developing germ cells [16, 17]. Therefore, the main approach exploring new roles for estrogens in male reproduction focuses on physiological inactivation of ERs by administrating selective modulators. Clomiphene is a member of the selective estrogen receptor modulator (SERM) family that shows both estrogen agonist as well as antagonistic properties, acting as an agonist when the hormone is in low concentration and as an antagonist at high estrogen concentrations [18]. Clomiphene induces the ovulation in infertile women and stimulates the gonadotropin release in infertile oligospermic males [19].

Vertebrate seasonal breeders are an excellent model study to understand the spermatogenic mechanisms underlying spermatogenesis. In particular in reptiles, the cellular timing inside the seminiferous epithelium and the epididymal morphology both closely related to hormonal fluctuations allows to shed light on the mechanism controlling the regular progression of spermatogenesis. In this class of vertebrates, the mating period occurs generally in spring when testosterone determines the production of a huge amount of spermatozoa that move from the testis to the epididymis to be ejaculated. At the end of the summer season, the estrogen increases and the mating period stops. In this period, the seminiferous tubules show only spermatogonia and Sertoli cells. At the beginning of the new year, spermatogenesis starts again to culminate in spring with the onset of a new mating period [20, 21]. Though in reptiles spermatogenesis follows a cyclical trend, the testis structure is very similar to that of mammals, including the function accomplished by somatic cells. In the freshwater turtle *Kinosternon scorpioides*, for example, the Sertoli cell efficiency, that is, the number of round spermatids for Sertoli cell, is a determinant for sperm production as established for mammals [22]. Studies carried out on the lizard *Podarcis sicula* [21] also demonstrated that the regulation of the spermatogenic cycle is closely related to androgen and estrogen receptors, as in mammals.

In this frame, the aim of our study was to investigate the interference of Clomiphene citrate, as SERM member, during spermatogenesis in the seasonal breeder *Podarcis sicula*. To deepen a possible involvement of estrogen in the process, we have also compared the results with those obtained treating the animals with FSH or with FSH-Clomiphene.

By means of morphological and immunohistochemical investigations, we examined the structure of the testis and epididymis and the ER*α* distribution during the mating season, in the presence of Clomiphene alone or in combination with FSH. Exploiting the ability of *P. sicula* male liver to produce vitellogenin (VTG) and ER*α* under estrogen induction [9, 23–26], we also determined whether FSH or Clomiphene is able to induce an estrogen-like response, carrying out immunohistochemical investigations on liver sections with anti-ER*α* or anti-VTG antibodies.

## 2. Material and Methods

### 2.1. Animals and Experimental Treatments

Adult males of lizard *Podarcis sicula* of the field of origin (about 7.5–8 cm snout-vent) were caught in the outskirts of Naples (Italy) during the mating period (March–May) (*n* = 36) and kept in terrariums at natural temperature and photoperiod, fed ad libitum with larvae of *Tenebrio molitor*.

For Clomiphene treatment, animals (*n* = 8) received an intraperitoneal injection of Clomiphene citrate (Sigma-Aldrich, mixture of *cis*- and *trans*-isomers; 2.5 *μ*g/body weight) in reptile physiological solution (NaCl 0.07‰) every second day for two weeks. For FSH treatment, *P. sicula* samples (*n* = 8) received an intraperitoneal injection of FSH (Sigma-Aldrich; 30 *μ*g/body weight) in reptile physiological solution (NaCl 0.07‰) every second day for two weeks.

For FSH-Clomiphene treatment, the animals (*n* = 8) received an intraperitoneal injection of FSH (30 *μ*g/body weight) in reptile physiological solution (NaCl 0.07‰) every second day for two weeks and after this period, they received an intraperitoneal injection of Clomiphene citrate (2.5 *μ*g/body weight) for two weeks.

The Clomiphene and FSH concentrations used in this research correspond to the lowest doses which able to elicit an estrogenic effect in male lizard, as demonstrated by the VTG synthesis in the liver.

As controls, four untreated animals were sacrificed immediately after the capture, whereas eight animals were injected with the physiological saline solution every second day and sacrificed after 2 (*n* = 4) and 4 (*n* = 4) weeks of treatment.

All the animals were killed by decapitation after deep anaesthesia with ketamine hydrochloride (Parke-Davis, Berlin, Germany), 325 *μ*g/g body weight; the testis, epididymis, and liver were quickly removed and immediately processed for the histological analyses. All the experiments were approved and carried out in compliance with the ethical provisions enforced by the National Committee of the Italian Ministry of Health on in vivo experimentation (Department for Veterinary Public Health, Nutrition and Food Safety, SCN/2D/2000/9213) and organized to minimize animals number and suffering.

### 2.2. Histology

The testes with attached epididymides and livers were fixed in Bouin's fluid and processed for paraffin wax embedding according to routine protocols. Sections of 7 *μ*m in thickness were obtained with Reichert-Jung 2030 microtome. Sections were stained with haemalum/eosin to show general morphology, with Mallory's trichrome modified by Galgano [27], to view connective tissue fibers, or used for immunohistochemistry (IHC). All the histological results were examined by using a Nikon-MicroPhot-FXA light microscope.

### 2.3. Immunohistochemistry

For immunostaining, sections of the testis and liver previously fixed in Bouin's solution were deparaffinised and rehydrated as described [28], then washed in PBS, microwaved at 750 W for 15 min in citrate buffer, washed in 0.1% bovine serum albumin in PBS, and incubated with primary anti-hER*α* antibody (1 : 80) (Novocastra, United Kingdom) or homologous anti-VTG (1 : 1000) antibody [23, 29] in phosphate buffer 0.1 M pH 7.4 overnight at 4°C. The antigens were identified as previously reported [25] with Novolink Max Polymer Detection System (Leica Biosystems) according to the manufacturer's procedure. Negative controls of reactions were performed on other sections by omitting the primary antibodies in the incubation mixture.

## 3. Results

The testis, epididymis, and liver from the untreated animals showed the same features, regardless of the time of sacrifice and the administration of the physiological solution; hence from now on, they will be indicated as controls.

### 3.1. Clomiphene and FSH Effects on Testis and Epididymis Morphology

**Figure 1:**
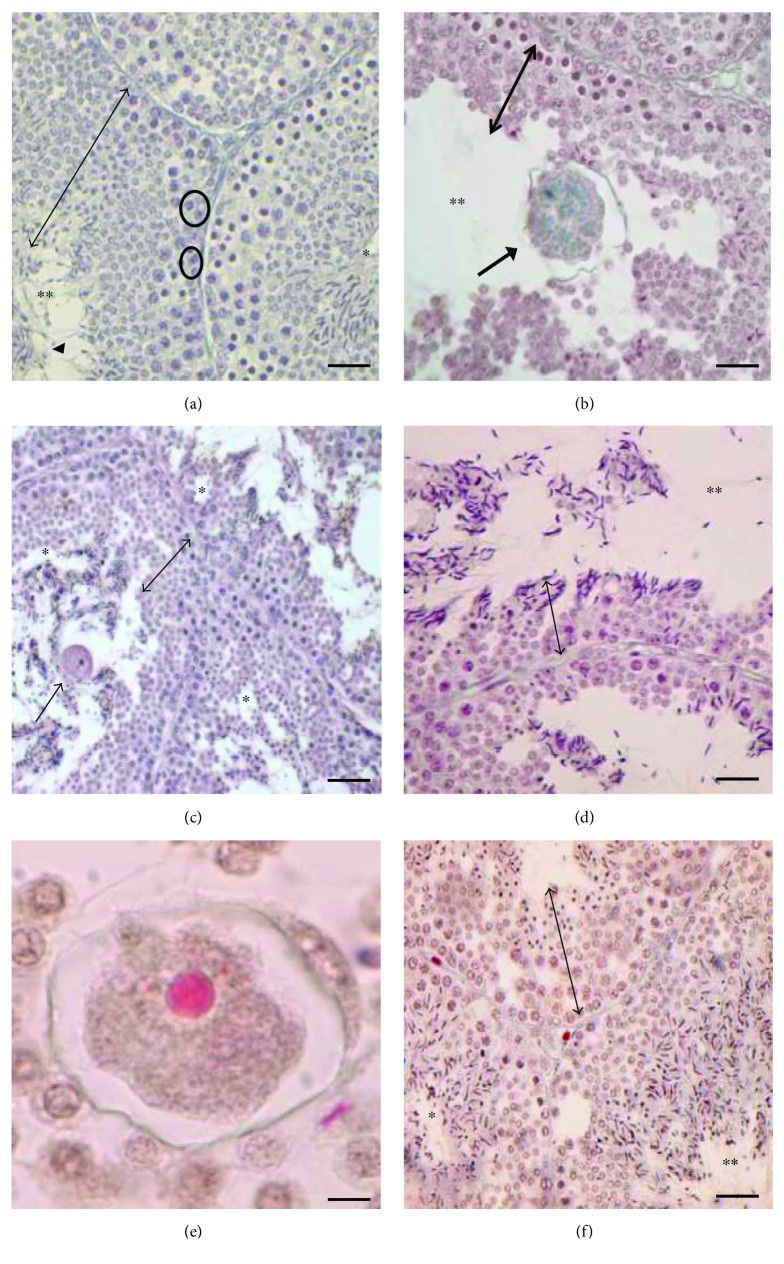
Histology of *P. sicula* testis. (a) Untreated males, mating period: the seminiferous epithelium is thick (↕) and full of germ cells in all spermatogenic stages, from spermatogonia (○) at the basis of the tubules to spermatozoa (◄) in the restricted lumen (∗∗). (b) Clomiphene-treated samples: the seminiferous epithelium is reduced in thickness (↕); in the lumen, some degenerate cells and oocyte-like structures are evident (↑). (c, d) FSH-treated samples: in the thin seminiferous epithelium, few germ cells and several empty spaces (∗) are present, and oocyte-like structures (↑) are also evident. (e) Detail at high magnification of oocyte-like structure. (f) FSH-Clomiphene-treated samples: all stages of spermatogenesis are evident and the epithelium thickness is increased (↕). The bar is 30 *μ*m.

**Figure 2:**
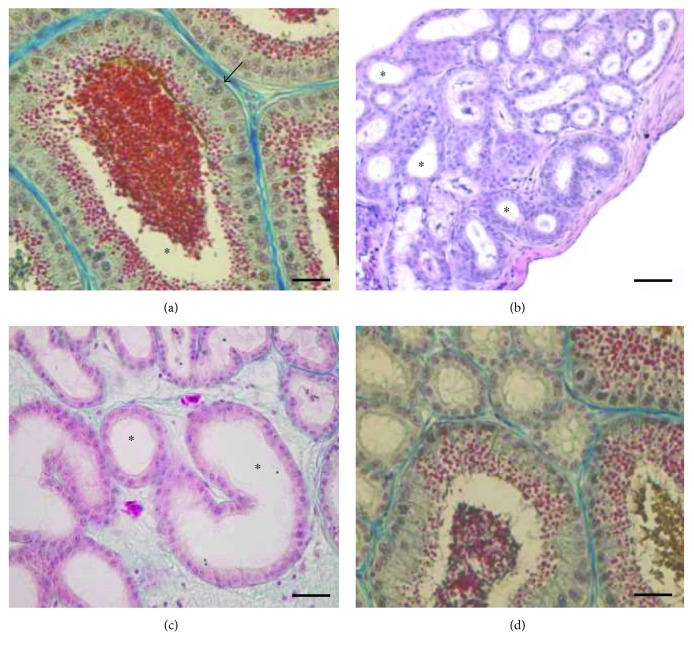
Histology of *P. sicula* epididymis. (a) Untreated males, mating period: the epithelium of the *corpus* is elongated and secreting and a large amount of granules is evident inside the lumen (∗). (b) Clomiphene-treated samples: the cells of the corpus are not secreting; in the lumen, no spermatozoa and secretory granules are evident (∗). (c) FSH-treated samples: in the lumen of the *corpus*, no granules and spermatozoa are evident (∗). (d) FSH-Clomiphene-treated samples: the cells lining the *corpus* are secreting and some granules and spermatozoa are evident in the lumen (∗). The bar is 30 *μ*m.

The testes of lizards collected during the mating period showed the seminiferous epithelium full of germ cells in all the stage of differentiation, from spermatogonia to spermatozoa (Figure 1(a)). The epididymis in this period is in full activity, the epithelium of the *corpus* showed cylindrical and elongated cells with a large amount of secretory granules inside, and the enlarged lumen of the *corpus* was full of spermatozoa and secretory granules (Figure 2(a)).

In the animals injected with Clomiphene, the seminiferous epithelium was reduced in thickness (Figure 1(b)), and the lumen of the tubules was wide and partially occupied by oocyte-like structures (Figures 1(b) and 1(e)). The epididymis in these animals was regressed: the epithelial cells lining the *corpus* were flattened and not secreting without spermatozoa and secretory granules in the lumen (Figure 2(b)).

In the lizards treated with FSH, the testis morphology resembled as that observed in Clomiphene-treated animals: the seminiferous epithelium was thin and formed by few germ cells; oocyte-like structures were detected in the lumen (Figures 1(c) and 1(d)). Also, the epididymis showed some regression signals, in particular, the epithelial cells lining the *corpus* were poorly secreting, and very few spermatozoa and secretory granules were present in the lumen (Figure 2(c)).

Conversely, in the lizards treated with FSH followed by the Clomiphene exposure, the morphology of the testis was similar to that observed in control animals, showing a thick seminiferous epithelium with germ cells in all the spermatogenic stages (Figure 1(f)). The epididymis was active, the epithelial cells of the *corpus* were secreting, and spermatozoa and granules were evident in the lumen (Figure 2(d)), albeit lower than in controls (Figure 2(a)).

### 3.2. Detection of ER*α* and VTG Protein by Immunohistochemistry

#### 3.2.1. Testis

**Figure 3:**
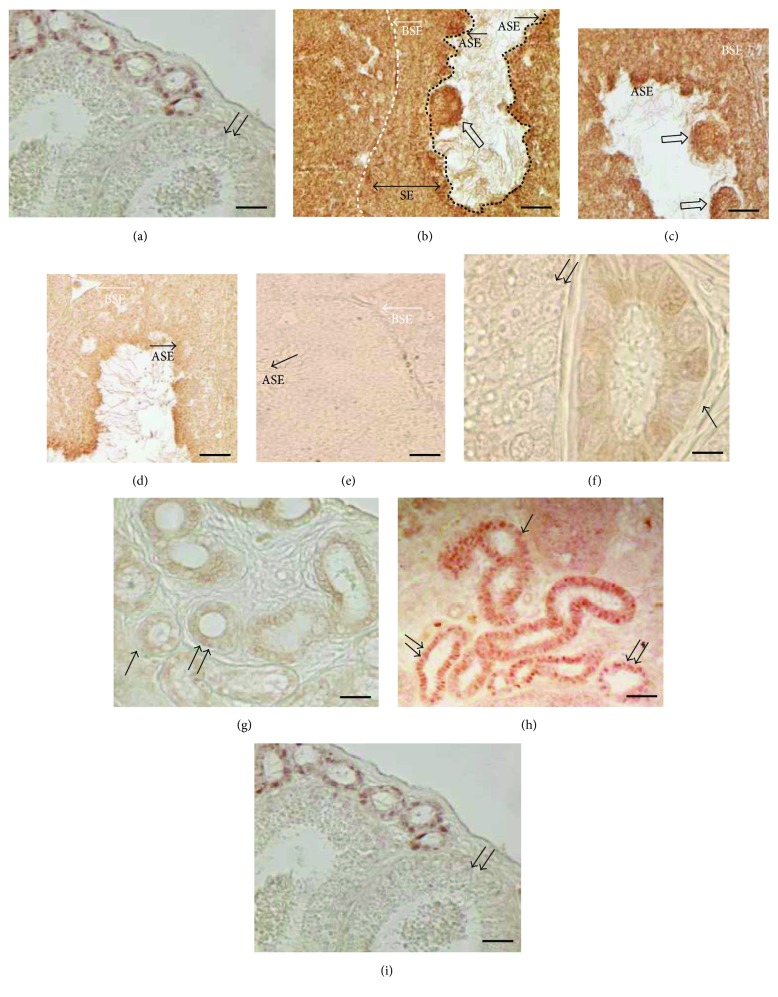
Immunohistochemistry with ER*α* antibody on the testis (a–e) and epididymis (f–i). (a) Untreated males: ir-ER*α* is evident only in the final differentiating stage, that is, spermatozoa. (b) Clomiphene-treated and (c) FSH-treated samples: ir-ER*α* is present in all the cells inside the seminiferous epithelium (SE). The dotted white and black lines indicate the basal (BSE) and apical (ASE) seminiferous epithelium, respectively. Note in (b, c), oocyte-like structure marked for ER*α* (⇧). (d) FSH-Clomiphene-treated samples: ir-ER*α* is evident only in spermatozoa. (f) Untreated males: ir-ER*α* is absent in the *corpus* (↑↑) but present in the *efferent ductules* (↑). (g) Clomiphene-treated and (h) FSH-treated samples: ir-ER*α* is evident in the cells lining the *corpus* (↑↑) and in the *efferent ductules* (↑). (i) FSH-Clomiphene-treated samples: ir-ER*α* is absent in the *corpus* (↑↑). (e) Negative control prepared omitting the antibody in the reaction. The bar is 30 *μ*m.

In control animals, immunoreactive-ER*α* (ir-ER*α*) was detected only in spermatozoa, and no immunoreactivity was evident in the other germ cells forming the seminiferous epithelium (Figure 3(a)).

In the Clomiphene-treated animals, as well as in FSH-treated lizards, ir-ER*α* was evident in all the few spermatogonia, spermatocytes, spermatids, and spermatozoa forming the particularly thin seminiferous epithelium (Figures 3(b) and 3(c)). The oocyte-like structures in the lumen were also positive for ER*α* (Figure 3(b)).

In the lizards treated with FSH followed by Clomiphene exposure, ir-ER*α* was evident only in germ cells facing the lumen of the tubules, that is, spermatozoa, and no positivity was recorded in the other cells of the epithelium (Figure 3(d)), as observed in controls (Figure 3(a)).

The negative control of reaction obtained by omitting primary anti-ER*α* antibody on twin serial sections always gave no immunoreactive signals (Figure 3(e)).

#### 3.2.2. Epididymis

As expected in the mating period [20], in the epididymal tract of *P. sicula*-untreated males, IHC investigations showed that only the *corpus* appeared devoid of ER*α* immunoreactivity, while the *efferent ductules* and *cauda* were positive to ir-Er*α* signal (Figure 3(f)).

In the samples from specimens treated with Clomiphene or FSH, a massive presence of ER*α* was evident in the *corpus*, and the *efferent ductules* and *cauda* were always positive (Figures 3(g) and 3(h)).

Finally, in the animals injected first with FSH and then with Clomiphene, no positivity was recorded in the cells of the *corpus* while a strong positivity to anti-ER*α* antibody was still evident in the *efferent ductules* and *cauda* (Figure 3(i)), as observed in control animals.

The control of reaction obtained by omitting primary anti-ER*α* antibody on twin serial sections always gave negative results (data not shown).

#### 3.2.3. Liver

To assess the ability of FSH or Clomiphene to induce an estrogen-like response in this lizard, IHC analysis was carried out with ER*α* or VTG antibodies on sections of the liver from the animals in all the different experimental conditions.

**Figure 4:**
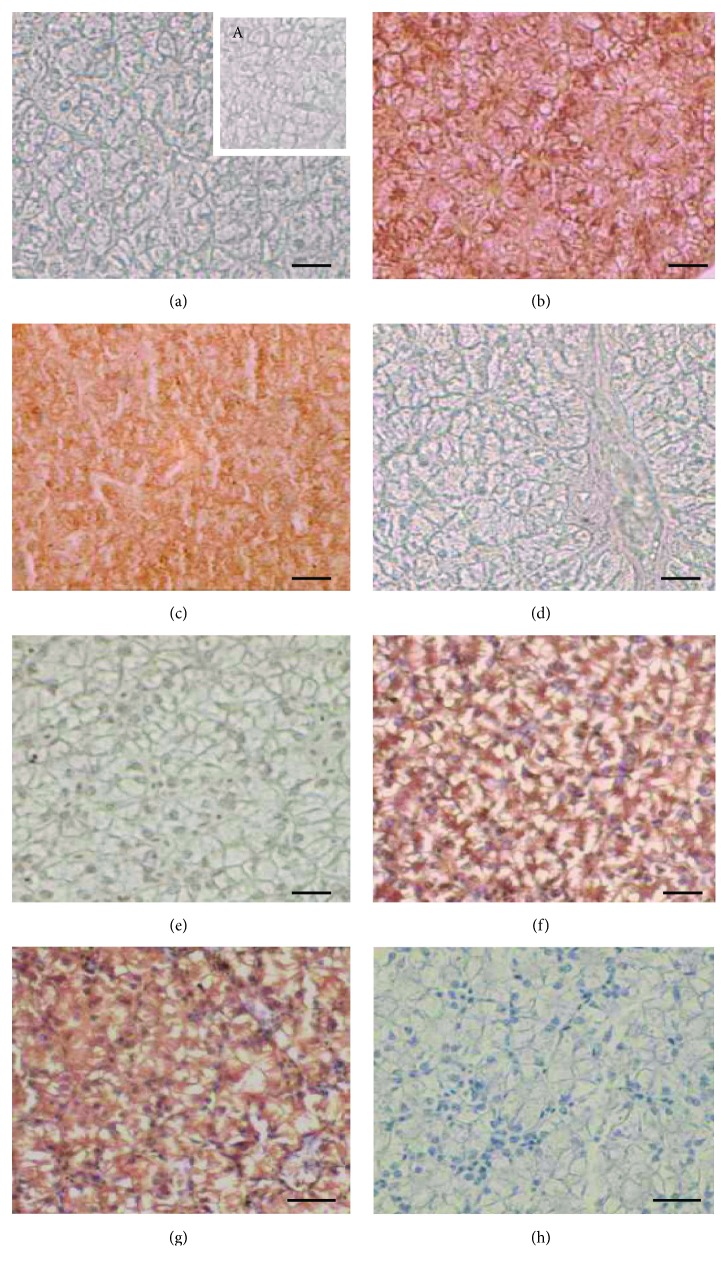
Immunohistochemistry with ER*α* (a–d) or VTG (bottom panel, e–f) antibodies in the liver. (a) Untreated males: no immunoreactivity is detected to anti-ERα antibody; the box (A) shows the negative control of reaction, performed lacking the antibody. (b) Clomiphene-treated and (c) FSH-treated samples: ir-ER*α* is evident in cells. (d) FSH-Clomiphene: no immunoreactivity is detected as in untreated samples (a). (e) Untreated males: no immunoreactivity is detected to anti-VTG antibody. (f) Clomiphene-treated and (g) FSH-treated samples: ir-VTG is evident in the cytoplasm cells. (h) FSH-Clomiphene: no immunoreactivity is detected, as in untreated samples (e). In (e), (f), (g), and (h), cell nuclei were counterstained with Mayer's haemalum. The bar is 30 *μ*m.

In the liver of control specimens, no positivity to ER*α* or VTG was evident (Figures 4(a) and 4(e)), as expected in males [23].

In Clomiphene- or FSH-treated animals, liver cells showed immunoreactivity for both ER*α* (Figures 4(b) and 4(c)) and VTG (Figures 4(f) and 4(g)).

In the liver of males treated with FSH followed by Clomiphene exposure, no positivity to ER*α* (Figure 4(d)) neither VTG antibodies (Figure 4(h)) was detected.

The negative control sections incubated without primary antibodies were devoid of reaction (Figure 4(a), A).

## 4. Discussion

This research analyzes for the first time the estrogenic-mediated effects of Clomiphene or/and FSH treatment on the spermatogenesis and epididymis activity in the lizard *Podarcis sicula*.

In the seasonal breeder *Podarcis sicula*, during the mating period, the seminiferous epithelium shows all the developmental stages of germ cells and a great amount of sperms fills the lumen. Parallelly, the epididymis is fully active, with the cells of the *corpus* high and secreting [20, 30, 31]. In reptiles, very scant is the knowledge about the effects of SERM exposition in both males and females. In *P. sicula*, the effect of tamoxifene to restore the estradiol-induced increase of mast cells number in the testis and in Harderian gland was investigated [32, 33]. In *Anolis carolinensis*, the enclomiphene and zuclomiphene were ineffective to stimulate sexual receptivity in ovariectomized female when given alone or as priming regimen prior to E2 [34].

First, our results demonstrate that Clomiphene or FSH exposures during mating period affect the morphology of the testis and epididymis. In particular, the slowdown of spermatogenesis, the presence of oocyte-like cells in the seminiferous epithelium, and the reduced secretive activity of the epididymal *corpus* represent the classical alterations due to estrogenic exposure, already described in this lizard [9, 10, 20].

Differently, in mammals and in particular in men with low testosterone levels, it has been demonstrated that Clomiphene is able to restore spermatogenesis through the rising of endogenous serum FSH, LH, and testosterone levels [35, 36].

In order to ascertain the estrogen-like action of Clomiphene or FSH, we performed immunohistochemistry investigations on two estrogen-responsive proteins in the liver, that is, VTG and ER*α*. It is known in fact that in *P. sicula* male liver, VTG and ER*α* genes are silent and can be activated exclusively under estrogenic stimulation; on the contrary, ER*β* in this tissue is constitutively expressed [23–25]. Hence, the presence of ER*α* and VTG in the liver of Clomiphene- or FSH-treated males represents the unequivocal evidence of the ability of these two substances to create estrogenic milieu into the organism.

It has been demonstrated that Clomiphene, through ERα, acts as estrogen agonist when the circulating concentration of the hormone is low [18]. Since in the mating period the levels of circulating estrogens in male *P. sicula* are low [30], it is conceivable that the morphological alterations now observed in the testis and epididymis are due to the estrogenic environment caused by Clomiphene.

As regarding the estrogenic effect induced by FSH, it is known that this hormone is able to stimulate aromatase expression in mammalian granulosa cells [37–39]. It is possible that in male *P. sicula*, FSH increases the amount of circulating estrogen according to a cascade pathway: stimulating the aromatase activity, it enhances estradiol secretion that in turn stimulates the synthesis of its receptor resulting in an amplification of the estrogenic signal [40, 41]; in parallel, the increased conversion of the androgens in estrogens carried out by the aromatase could cause a testosterone reduction with the consequent spermatogenesis slowdown.

Exploiting the ability of Clomiphene to act also as an estrogen antagonist when a high amount of hormone is present [18], we decided to treat the FSH-exposed animals with Clomiphene to evaluate the effect of this substance after the FSH-induced estrogen increase. The results show that Clomiphene, after FSH injections, is able to restore in male gonad a condition comparable to that observed in control animals, typical of the full mating period. The lack of ER*α* and VTG protein in the liver of FSH-Clomiphene animals reinforces the data obtained by the morphological observation on the antagonistic estrogenic property of Clomiphene and confirmed its ability to restore the initial basal condition in male gonad.

The estrogen responsiveness of *P. sicula* testis and epididymis to Clomiphene and FSH has been also demonstrated by immunohistochemistry analysis with ER*α* antibody.

As regarding the presence of the estrogen receptors in these two organs, it is known that ER*β* shows a widespread expression and synthesis, whereas ER*α*-mRNA undergoes fluctuations during the reproductive cycle [21, 42]. In particular, it has been demonstrated that ER*α* switches off the secretory activity of the epididymal *corpus* both in nonreproductive period and after estrogen treatment; ER*β* expression does not undergo any changes remaining always constitutively expressed, as observed in the liver [20]. Hence, the presence of ER*α* protein in nonsecreting epididymal *corpus* of Clomiphene- or FSH-treated animals and its absence in the secreting cells of the *corpus* of FSH-Clomiphene animals demonstrate that both Clomiphene and FSH interfere with the estrogen signalling through the modulation of ER*α*.

In the testis, the results demonstrate that in the mating period, under natural conditions, ER*α* is present only in spermatozoa, although the mRNA was been found in all germ cells [21]; after Clomiphene or FSH treatment, ER*α* immunoreactivity is detected in all the few germ cells present inside the tubules. This may be due to the fact that estrogen are able to induce the synthesis of ER*α* leading to hormonal imbalance that threatens the functionality of the testis [10, 43, 44]. On the other hand, in FSH-Clomiphene samples, ir-ER*α* is confined only in spermatozoa, as observed in the natural animals of the mating period.

## 5. Conclusions

Taken together, our investigations display the ability of the testis and epididymis of the lizard *Podarcis sicula* to control their morphophysiology by regulating the intracellular presence of ER*α*. The results also add new information on the requirements of male gonads in relation to the estrogenic environment, underlining once again the importance of the traditionally recognized as sex female hormone estrogen for the right spermatogenesis progression.

## References

[1] Parvinen M. (1982). Regulation of the seminiferous epithelium. *Endocrine Reviews*.

[2] De Gendt K., Verhoeven G. (2012). Tissue- and cell-specific functions of the androgen receptor revealed through conditional knockout models in mice. *Molecular and Cellular Endocrinology*.

[3] Nicholls P. K., Stanton P. G., Chen J. L. (2012). Activin signaling regulates Sertoli cell differentiation and function. *Endocrinology*.

[4] Pitetti J. L., Calvel P., Zimmermann C. (2013). An essential role for insulin and IGF1 receptors in regulating Sertoli cell proliferation, testis size, and FSH action in mice. *Molecular Endocrinology*.

[5] Crespo D., Assis L. H. C., Furmanek T., Bogerd J., Schulz R. W. (2016). Expression profiling identifies Sertoli and Leydig cell genes as Fsh targets in adult zebrafish testis. *Molecular and Cellular Endocrinology*.

[6] Shiraishi K., Matsuyama H. (2017). Gonadotoropin actions on spermatogenesis and hormonal therapies for spermatogenic disorders [review]. *Endocrine Journal*.

[7] Sharpe R. M. (1998). The roles of oestrogen in the male. *Trends in Endocrinology and Metabolism*.

[8] Carreau S. (2000). Estrogens and male reproduction. *Folia Histochemica et Cytobiologica*.

[9] Verderame M., Limatola E. (2015). Interferences of an environmental pollutant with estrogen-like action in the male reproductive system of the terrestrial vertebrate *Podarcis sicula*. *General and Comparative Endocrinology*.

[10] Verderame M., Limatola E., Scudiero R. (2016). Ectopic synthesis of vitellogenin in testis and epididymis of estrogen-treated lizard *Podarcis sicula*. *General and Comparative Endocrinology*.

[11] Eddy E. M., Washburn T. F., Bunch D. O. (1996). Targeted disruption of the estrogen receptor gene in male mice causes alteration of spermatogenesis and infertility. *Endocrinology*.

[12] Hess R. A., Bunick D., Lee K. H. (1997). A role for oestrogens in the male reproductive system. *Nature*.

[13] Hess R. A., Gist D. H., Bunick D. (1997). Estrogen receptor (α and β) expression in the excurrent ducts of the adult male rat reproductive tract. *Journal of Andrology*.

[14] Robertson K. M., O'Donnell L., Jones M. E. E. (1999). Impairment of spermatogenesis in mice lacking a functional aromatase (*cyp 19*) gene. *Proceedings of the National Academy of Sciences of the United States of America*.

[15] Oliveira C. A., Carnes K., Franca L. R., Hess R. A. (2001). Infertility and testicular atrophy in the antiestrogen treated adult male rat. *Biology of Reproduction*.

[16] Chaki S. P., Misro M. M., Gautam D. K., Kaushik M., Ghosh D., Chainy G. B. (2006). Estradiol treatment induces testicular oxidative stress and germ cell apoptosis in rats. *Apoptosis*.

[17] Kaushik M. C., Misro M. M., Sehgal N., Nandan D. (2010). AR versus ER (α) expression in the testis and pituitary following chronic estrogen administration in adult rat. *Systems Biology in Reproductive Medicine*.

[18] Kurosawa T., Hiroi H., Momoeda M., Inoue S., Taketani Y. (2010). Clomiphene citrate elicits estrogen agonistic/antagonistic effects differentially via estrogen receptors α and β. *Endocrine Journal*.

[19] Kamath M. S., George K. (2011). Letrozole or clomiphene citrate as first line for anovulatory infertility: a debate. *Reproductive Biology and Endocrinology*.

[20] Verderame M., Angelini F., Limatola E. (2012). Expression of estrogen receptor alpha switches off secretory activity in the epididymal channel of the lizard *Podarcis sicula*. *Molecular Reproduction & Development*.

[21] Verderame M., Angelini F., Limatola E. (2014). Spermatogenic waves and expression of AR and ERs in germ cells of *Podarcis sicula*. *International Journal of Zoology*.

[22] Sousa A. L., Campos-Junior P. H., Costa G. M., de Franca L. R. (2014). Spermatogenic cycle length and sperm production in the freshwater turtle *Kinosternon scorpioides*. *Biology of Reproduction*.

[23] Verderame M., Limatola E. (2010). Molecular identification of estrogen receptors (*ERα* and *ERβ*) and their differential expression during VTG synthesis in the liver of lizard *Podarcis sicula*. *General and Comparative Endocrinology*.

[24] Verderame M., Prisco M., Andreuccetti P., Aniello F., Limatola E. (2011). Experimentally nonylphenol-polluted diet induces the expression of silent genes VTG and ERα in the liver of male lizard *Podarcis sicula*. *Environmental Pollution*.

[25] Verderame M., Limatola E., Scudiero R. (2016). Estrogenic contamination by manure fertilizer in organic farming: a case study with the lizard *Podarcis sicula*. *Environmental Toxicology*.

[26] Verderame M., Scudiero R. (2017). Estrogen-dependent, extrahepatic synthesis of vitellogenin in male vertebrates: a mini-review. *Comptes Rendus Biologies*.

[27] Mazzi A. (1977). *Manuale di tecniche istologiche e istochimiche*.

[28] Agnese M., Valiante S., Rosati L., Andreuccetti P., Prisco M. (2016). Pituitary adenylate cyclase-activating peptide (PACAP) and PAC1 receptor in the testis of cartilaginous fish *Torpedo marmorata*: a molecular and phylogenetic study. *Comparative Biochemistry and Physiology Part B: Biochemistry and Molecular Biology*.

[29] Del Giudice G., Prisco M., Agnese M. (2012). Effects of nonylphenol on vitellogenin synthesis in adult males of the spotted ray *Torpedo marmorata*. *Journal of Fish Biology*.

[30] Angelini F., Botte V., Dallai R. (1992). Spermatogenesis in reptiles, dynamic and regulatory aspect. *Sex Origin and Evolution*.

[31] Verderame M. (2014). The involvement of the androgen receptor in the secretion of the epididymal *corpus* in the lizard *Podarcis sicula*. *International Journal Zoology*.

[32] Minucci S., Vitiello I. I., Marmorino C., di Matteo L., Baccari G. C. (1995). Mast cell-Leydig cell relationships in the testis of the lizard *Podarcis s. sicula* Raf: thermal manipulation, ethane 1,2-dimethane sulphonate (EDS) and sex hormone treatment. *Zygote*.

[33] Vitiello I. I., Baccari G. C., di Matteo L., Rusciani A., Chieffi P., Minucci S. (1997). Number of mast cells in the Harderian gland of the lizard *Podarcis sicula sicula* (Raf): the annual cycle and its relation to environmental factors and estradiol administration. *General and Comparative Endocrinology*.

[34] Tokarz R. R., Crews D. (1982). Failure of a variety of antiestrogens to mimic estrogen action in the induction of sexual receptivity in a female lizard. *Hormones and Behavior*.

[35] Kaminetsky J., Werner M., Fontenot G., Wiehle R. D. (2013). Oral enclomiphene citrate stimulates the endogenous production of testosterone and sperm counts in men with low testosterone: comparison with testosterone gel. *The Journal of Sexual Medicine*.

[36] Patankar S. S., Kaore S. B., Sawane M. V., Mishra N. V., Deshkar A. M. (2007). Effect of clomiphene citrate on sperm density in male partners of infertile couples. *Indian Journal of Physiology and Pharmacology*.

[37] Fitzpatrick S. L., Richards J. S. (1991). Regulation of cytochrome P450 aromatase messenger ribonucleic acid and activity by steroids and gonadotropins in rat granulosa cells. *Endocrinology*.

[38] Silva J. M., Price C. A. (2002). Insulin and IGF-I are necessary for FSH induced cytochrome P450 aromatase but not cytochrome P450 sidechain cleavage gene expression in oestrogenic bovine granulose cells in vitro. *Journal of Endocrinology*.

[39] Silva J. M., Hamel M., Sahmi M., Price C. A. (2006). Control of oestradiol secretion and of cytochrome P450 aromatase messenger ribonucleic acid accumulation by FSH involves different intracellular pathways in oestrogenic bovine granulosa cells *in vitro*. *Reproduction*.

[40] Pakdel F., Delaunay F., Ducouret B. (1997). Regulation of gene expression and biological activity of rainbow trout estrogen receptor. *Fish Physiology and Biochemistry*.

[41] Custodia-Lora N., Novillo A., Callard I. P. (2004). Effect of gonadal steroids on progesterone receptor, estrogen receptor, and vitellogenin expression in male turtles (*Chrysemys picta*). *Journal of Experimental Zoology*.

[42] Chieffi P., Varriale B. (2004). Estrogen receptor β localization in the lizard (*Podarcis s. sicula*) testis. *Zygote*.

[43] Kinnberg K., Korsgaard B., Bjerregaard P. (2000). Concentration-dependent effects of nonylphenol on testis structure in adult platyfish *Xiphophorus maculatus*. *Marine Environmental Research*.

[44] Christiansen T., Korsgaard B., Jespersen Å. (1998). Effects of nonylphenol and 17 beta-oestradiol on vitellogenin synthesis, testicular structure and cytology in male eelpout Zoarces viviparous. *The Journal of Experimental Biology*.

